# Treatment of perioperative swelling by rest, ice, compression, and elevation (RICE) without and with additional application of negative pressure (RICE^+^) in patients with a unilateral ankle fracture: study protocol for a monocentric, evaluator-blinded randomized controlled pilot trial

**DOI:** 10.1186/s40814-021-00944-7

**Published:** 2021-11-12

**Authors:** Dagmar-C Fischer, Axel Sckell, Angelina Garkisch, Klaus Dresing, Anton Eisenhauer, Luzia Valentini, Thomas Mittlmeier

**Affiliations:** 1 Department of Pediatrics, Rostock University Medical Center, Ernst-Heydemann-Str. 8, 18057 Rostock, Germany; 2 Department of Traumatology, Hand and Reconstructive Surgery, Rostock University Medical Center, Rostock, Germany; 3 Department of Trauma Surgery, Orthopaedics and Plastic Surgery, University Medicine Göttingen, Georg-August-University, Göttingen, Germany; 4 GEOMAR Helmholtz Center for Ocean Research Kiel, Kiel, Germany; 5 Department of Agriculture and Food Sciences, Neubrandenburg Institute for Evidence-Based Dietetics (NIED), University of Applied Sciences Neubrandenburg, Neubrandenburg, Germany

**Keywords:** Ankle fracture, Rest, Ice, Compression, Elevation, Edema, Postoperative swelling, Negative pressure therapy, Soft tissue, Osseous healing, Complications

## Abstract

**Background:**

Edema is commonly seen after surgical fixation of ankle fractures. Rest, ice, compression, and elevation (RICE) is an established combination to prevent swelling but hardly able to stimulate lymphatic resorption. Recently, an epicutaneously applied negative pressure suction apparatus (LymphaTouch®) has been introduced to stimulate lymphatic flow. While postoperative recovery, soft tissue, and osseous healing as well as functional outcome are probably linked to the amount of postoperative swelling, estimates on this relative to prevention (RICE) or prevention + stimulated resorption (RICE^+^) of fluid are scarce.

**Methods and analysis:**

This is a single-center, evaluator-blinded randomized pilot trial to investigate postoperative swelling in adults requiring surgical fixation of a closed unilateral ankle fracture. A total of 50 patients will be recruited and randomly assigned to RICE or RICE^+^ prior to surgery. All patients will undergo evaluator-blinded measurements of the ankle volume the day before surgery and subsequently from the evening of the 2nd postoperative day every 24 h until discharge. RICE will be initiated right after surgery and continued until discharge from the hospital in all patients. Additional application of negative pressure therapy (RICE^+^) will be initiated on the morning of the 2nd postoperative day and repeated every 24 h until the time of discharge from the hospital. Outcome measures are (i) the relative amount and the time course of the postoperative swelling, (ii) the demand for analgesic therapy (type and amount) together with the perception of pain, (iii) the rate of complications, and (iv) mobility of the ankle joint and the recovery of walking abilities during a 12-weeks follow-up period. Serum and urine samples taken prior to sugery and during postoperative recovery will allow to evaluate the ratio of naturally occurring stable calcium isotopes (δ^44/42^Ca) as a marker of skeletal calcium accrual.

**Ethics and dissemination:**

The protocol was approved by the institutional Ethics Committee (Rostock University Medical Center, Rostock, Germany) in accordance with the Declaration of Helsinki (approval number: A 2020-0092). The results of this study will be actively disseminated through scientific publications and conference presentations.

**Trial registration:**

DRKS, DRKS00023739. Registered on 14 December 2020

## Introduction

### Background and rationale

Ankle fractures account for approximately 9% of all fractures, and occur preferentially either in physically active adults or as an osteoporotic fracture in the elderly [1–4]. Any injury, either accidentally as with the fracture or, in addition, as with surgical fixation, causes the release of inflammatory mediators with concomitant extravasation of fluid, i.e., swelling which in turn induces pain and hampers mobility. Preoperative relief of the edema is required for tension-free wound closure while postoperative swelling bears the inherent risk of wound complications and infections. Thus, although resolving pre- and postoperative swelling is ultimately required, the well-established and widely used combination of rest, ice, compression, and elevation (RICE) prevents the formation of edema rather than stimulating lymphatic resorption. Manual lymphatic drainage is a well-introduced procedure for managing post-lymphadenectomy lymphedema via stimulating of lymph circulation [5, 6]. However, clinical studies on lymphatic treatments after orthopedic surgery of the lower extremities are rare, and the results are controversial. Moreover, protocols differ with respect to inclusion/exclusion criteria, outcome measures, treatment modalities, and timing [6–12].

Very recently, an epicutaneously applied negative pressure suction apparatus (LymphaTouch®, Helsinki, Finland) has been introduced and successfully been used for the reduction of pre- and postoperative swelling in patients undergoing unilateral surgery on the upper and lower extremity [8, 13]. It is appealing to fortify the classical RICE concept by additional stimulation of lymphatic flow (RICE^+^) after surgical fixation of a closed unilateral ankle fracture. We already established contact-less photooptical 3D-scanning as a reliable and highly standardized method for the determination of ankle volume [14]. Additional measures of short- and long-term outcome relative to postoperative care according to the RICE or RICE^+^ concept are pain, postoperative complications, mobility of the joint, and walking abilities. Quantitative pulse echography (Bindex®, Bone Index Finland Ltd., Kuopio, Finland) will allow for scoring of bone mineral density (normal, osteopenic or osteoporotic). The ratio of naturally occurring stable calcium isotopes (δ^44/42^Ca) in the serum and urine has been introduced as a marker of bone mineral balance [15, 16]. Thus far, this marker is preferentially used to monitor bone mineral loss rather than accrual. Like numerous other blood-born marker, serum calcium concentration depends on the time of blood sampling, i.e., samples taken before and after breakfast are likely to contain different amounts of calcium. Thus, standardized blood sampling is a major prerequisite to use calcium isotope measurement as a marker of skeletal health. Since standardized blood sampling in an out-patient setting is challenging, we decided to incorporate this as a small side project. Patients are fairly comparable with respect to the skeletal defect (unilateral ankle fracture), and blood sampling before breakfast is easily done in a hospital setting. Blood and urine samples will be taken on a voluntary base and we expect that δ^44/42^Ca will change according to the recruitment of calcium into the bone, i.e., allows to monitor the process of fracture healing during postoperative recovery and the follow-up period relative to baseline values prior to surgery.

## Methods/design

### Aim of the study

This monocentric, evaluator-blinded randomized controlled pilot-trial aims (i) to obtain an estimate on the standard deviation of the normalized ankle volume during postoperative recovery and (ii) to assess the feasibility of the proposed randomized allocation to the treatments and the suggested outcome measures. The latter include the demand for analgesics, the perception of pain, the rate of complications, the mobility of the joint, walking abilities, and skeletal calcium accrual together with bone mineral density. In summary, the results may broaden the knowledge on the interference between soft tissue swelling, functional outcome, and bone metabolism.

### Design and setting of the study

This is a single-center evaluator-blinded randomized controlled pilot trial with two parallel groups and a 1:1 allocation ratio of patients receiving surgical fixation of an unilateral ankle fracture. The study is going to be conducted at the Department of Traumatology, Hand and Reconstructive Surgery at Rostock University Medical Center. Starting from the 2nd postoperative day, there will be daily interventions for up to 10 days (i.e., the time of hospitalization). Repeated measures of outcome will be performed at baseline (prior to surgery), during the intervention period, and during the 12-week follow-up period. The study will be performed according to the ethical standards of the Institutional Ethics Committee (approval number: A 2020-0092) and in accordance with the Declaration of Helsinki.

## Participants, interventions, and outcomes

### Eligibility criteria

Adults (≥ 18 years of age) requiring surgical fixation of a closed unilateral ankle fracture (any fracture type scheduled for surgery) are eligible. Additional *inclusion criteria* are (i) a BMI of at least 16 kg/m^2^ and at a maximum 35 kg/m^2^, (ii) willingness to be randomized, and (iii) the ability to give written informed consent. Patients with open uni- or bilateral fractures, a history of secondary lymphedema due to tumoral disease during the preceding 5 years, dietary patterns other than omnivore or lacto-ovo-vegetarian (e.g., vegan, macrobiotic), and/or being incapable of adherence to follow-up examinations are excluded. Furthermore, pregnant women are not eligible. Eligible patients will receive detailed information on the study by trained physicians of our outpatient facility where preoperative assessment and preoperative necessary preparations for the surgical procedure take place when the indication for surgery has been secured. Prior to enrollment, all patients have to read and to sign the informed consent.

### Randomization

A researcher neither involved in clinical care or the day-to-day running of the trial will handle the randomization. Patients who gave written informed consent are assigned to either one of the treatment arms according to the results of a list-based block randomization procedure [17]. Only the person in charge to apply the device-based negative pressure therapy and the patient are informed on the assigned treatment, and both are instructed not to comment on the mode of therapy.

### Sample size justification

This pilot study is designed to obtain an estimate on the standard deviation of the normalized ankle volume during postoperative recovery and to assess the feasibility of both, the proposed randomized allocation to the treatment arms, and the suggested outcome measures. According to the recommended sample size for feasibility studies, we aim to enroll 25 participants per arm (50 patients in total) [18–21]. This number should be sufficient to allow the determination of variability.

### Intervention

Patients randomized to either treatment group will receive identical postoperative care, i.e., prescription of analgesics and the classical RICE concept to stimulate resorption of the postoperative swelling. The latter will be initiated right after surgery and continued until discharge from the hospital. Patients are advised to strictly adhere to the RICE concept in order to minimize variability inherent to this type of therapy. In particular, a positioning splint will be placed under the knee and the operated lower leg to elevate the ankle above heart level and ice packs will be provided for cooling during daytime according to the individual preference of the patient. The staff on the ward will not be notified on the treatment arm but asked to record the number of ice packs per day as a surrogate measure of both, the individual preference for cooling and adherence to the baseline therapy. Mobilization during the postoperative period and walking will be allowed with crutches and a lower leg orthosis under partial weight-bearing with 20 kg according to a personal training on a scale under supervision of a physiotherapist and without notification of the physiotherapist on the treatment arm. In patients allocated to RICE^+^, the device-based negative pressure therapy will be initiated on the morning of the 2nd postoperative day by a trained physician/medical student (last year of education) and repeated every 24 h until discharge from the hospital. The first treatment will be right after the wound dressing has been changed to a transparent and sterile film dressing (OPSITE® Post-OP visible, Smith & Nephew Woundcare, Hamburg, Germany). This wound dressing will be applied to all study patients and allow for constant monitoring of the wound and peri-wound area while eliminating the need to remove the dressing every time before the objective evaluation of the edema by 3D scanning and to ensure blinding of the staff with respect to the treatment arm. In all patients scheduled for RICE^+^, negative pressure therapy (30 min per session) will be applied by a trained physician/medical student in a standardized manner, i.e., starting at the supraclavicular fossa and following the lymphatic vessels down to the affected ankle. We use the same sequence as in manual lymphatic drainage, i.e., to increase lymph kinetic in normal lymphatics by mild mechanical stimuli as this augments the drainage across the lymphatic watershed. When starting manual lymphatic treatment at the inflow of the lymph into the venous system, edema fluid will pass through the already dilated area with successive decongestion of the lymphatic trunk [13, 22, 23]. The size of the treatment cup (60–80 mm diameter), the pressure (20–250 mmHg), and the frequency of vibration (20–90 Hz) will be adapted to individual needs (Table 1). To ensure blinding of all persons involved in postoperative care, the person in charge for the device-based negative pressure therapy will show up with the equipment at the pre-defined time anyway.

**Table 1:** Device-based application of negative pressure during postoperative recovery

Treatment area	Technique	Negative pressure [mmHg]	Work-to-rest ratio	Details	Duration
Supraclavicular fossa region (both sides)	Stationary	50–80	2.0s/50%	Bilateral treatment 3 pulsations on 5 spots/side	1 min
Inguinal area of the injured side	Stationary	50–100	2.0s/50%	3–5 pulsation on the same spot followed by movement to the next spot	1 min
Thigh circumferential	Stationary	50–150	2.0s/50%	1. Step: 3–5 pulsation on the same spot followed by movement to the next spot	4 min
	Sliding	50–150	2.0s/50%	2. Step: direction distal to proximal towards the inguinal lymph nodes	2 min
Knee anterior + posterior	Stationary	50-150	2.0s/50%	3-5 pulsation on same spot followed by movement to next spot	2 min
Lower thigh circumferential	Stationary	50-150	2.0s/50%	1. Step: 3–5 pulsation on the same spot followed by movement to the next spot	4 min
	Sliding	50–150	2.0s/50%	2. Step: direction distal to proximal	2 min
Foot dorsal and plantar	Stationary	50–150	2.0s/50%	3–5 pulsation on the same spot followed by movement to the next spot	2 min
Entire lower limb	Sliding	50–150	2.0s/50%	Direction distal to proximal, repeated	5 min
Inguinal area of the injured side	Stationary	50–150	2.0s/50%	3–5 pulsation on the same spot followed by movement to the next spot	1 min
Supraclavicular fossa region	Stationary	50–80	2.0s/50%	3–5 pulsations	1 min

Stationary technology: Decompression with pulsation or continuous pulsation is achieved by aa vertical pull on the tissue. The treatment cup is held at the same position for 3–5 pulsations before being moved to the next treatment area. Both areas overlap by about one third

Sliding technique: Decompression is achieved with pulsation or continuous adjustment of additional frequency changes to stimulate flow from the edematous tissue into the lymphatic system. The treatment cup is carefully slid over the skin, and a commonly used disinfectant will be applied to improve gliding

Work-to-rest ratio: The time of negative pressure application relative to the recovery time, i.e., length of the interval without negative pressure application

Patients of either treatment arm will be instructed not to comment on the mode of physical therapy, and the evaluator will have access neither to the ward during the times of treatment nor to the randomization list.

### Outcome measures

**Fig. 1:**
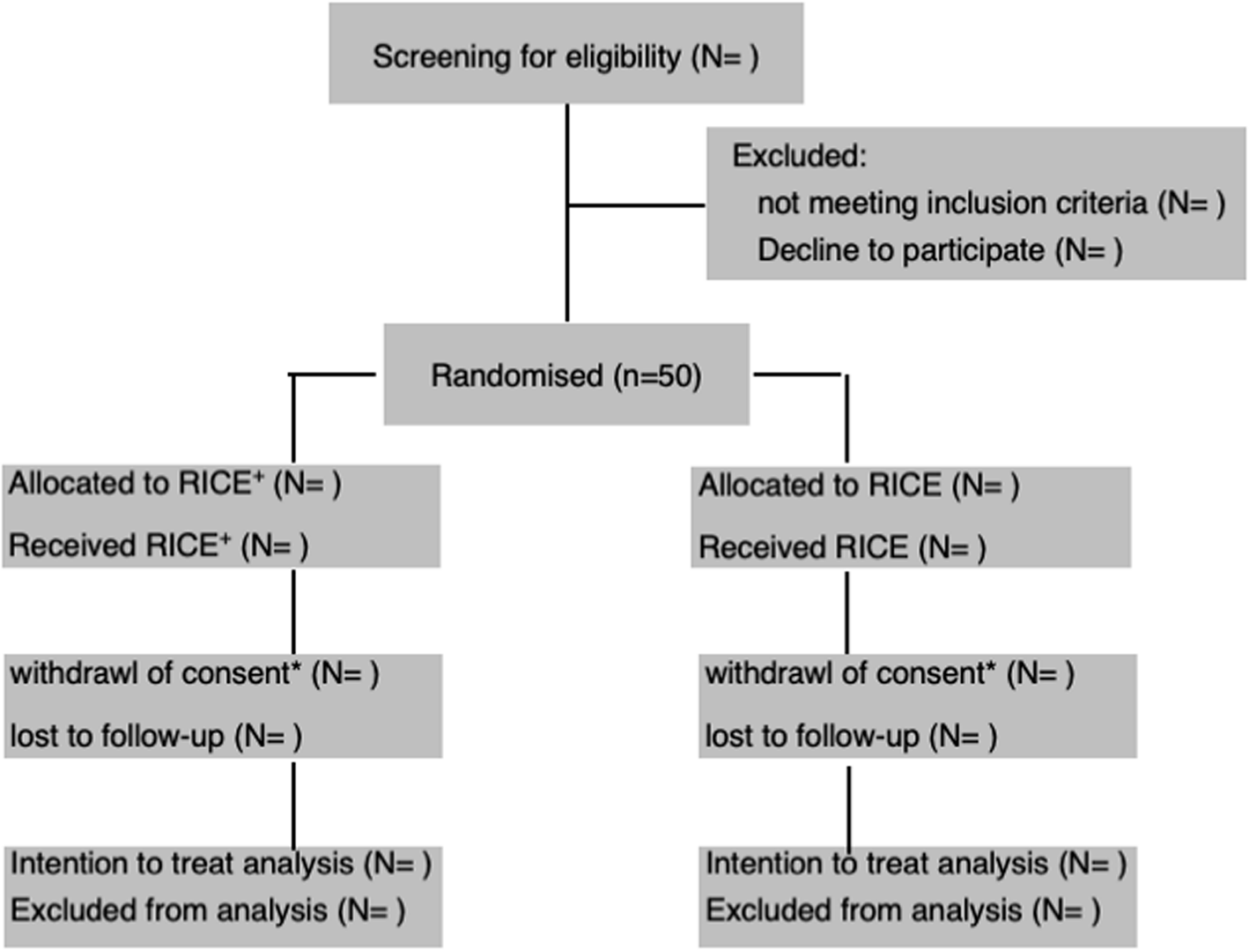
Study overview *reasons for withdrawl of consent and the number of drop-outs for evaluation of the primary and secondary endopoints are recorded.m

All measures will be taken at baseline and at pre-specified time points during the treatment and follow-up period (Fig. 1 and Table 2). Non-contact 3D scanning will be used to quantify postoperative swelling relative to the healthy/unaffected ankle by an evaluator blinded for the treatment. Scanning will start on the evening of the 2nd postoperative day. Subsequent measurements will be taken every 24 h until discharge from the hospital and by the same blinded evaluator. This data will allow to determine the variability of the normalized ankle volume and the kinetic of edema reduction (primary outcome). Secondary outcome measurements consist of the daily recordings of the individual pain perception by means of numeric analog scale, of the type and amount of analgesics, the quantification of the range of motion at both ankles (dorsi- and plantarflexion) with a goniometer at the time of discharge from the hospital and during follow-up visits. Walking abilities (factors of imbalance, ground reaction force) at the time of discharge from the hospital and during the follow-up period will highlight the functional outcome. Bone mineral density will be determined by means of quantitative pulse echography (Bindex®, Bone Index Finland Ltd., Kuopio, Finland) [24] at the time points indicated (Table 2). Sampling of blood and urine is on a voluntary base only, and these samples will be used to investigate the ratio of naturally occurring stable calcium isotopes (δ^44/42^Ca) in the serum and urine as a marker of bone mineral balance during fracture healing.

**Table 2:** Time schedule of the study examinations

	Screening	The day before surgery	Postoperative period starting day 2			Follow-up (weeks 2, 4, 6)	Final visit (week 12)
			Daily until discharge	Every 2nd day	Discharge		
Inclusion/exclusion criteria	x						
Informed consent	x						
Demographic and medical history	x						
Food Frequency Questionnaire ^17^		x				x^a^	x
Baseline laboratory test^b^		x			x		x
Serum/urine sampling^c^		x		x	x	x	x
Scoring of BMD^d^		x			x		x
Ankle swelling		x	x		x	x	x
Demand for analgesics		x	x		x	x	x
Pain perception (NAS)		x	x		x	x	x
Range of motion					x	x	x
Walking abilities					x	x	x

^a^Part of the follow-up examinations at week 4 only

^b^Routinely performed serum analysis (thyroid hormones, renal and liver function, clotting test, blood cell count) plus the determination of parathyroid hormone, 25-hydroxy vitamin D, total and ionized serum calcium, and phosphate

^c^Determination of δ^44/42^Ca (serum and urine), fetuin-A (serum), and FGF-23 (serum)

^d^Bone mineral density will be scored as normal, osteopenic, or osteoporotic by utilization of quantitative pulse echography (Bindex ®, Bone Index Finland Ltd., Kuopio, Finland)

#### Monitoring of the ankle volume during postoperative recovery in the hospital

The primary objective of the study is to investigate the amount of postoperative swelling relative to the application of RICE or RICE^+^. To ensure objective evaluation, a contact-less 3D optical scanning procedure (Artec®eva, Artec Studio, Luxembourg, Luxembourg) will be used to determine the volume of both ankles. To account for the interindividual variability of the ankle volume, per patient, the volume of the fractured ankle will be normalized to the one determined at the healthy site. The scan will cover an area ranging from the planta pedis up to one-third of the lower leg. The latter is defined as the distance between the medial knee joint space and the medial ankle. The upper border of the area to be scanned will be marked with permanent felt-tip pens. Proprietary software of the scanner (Artec studio V15, Artec3D, Luxembourg, Luxembourg) will be used to quantify ankle volumes. To minimize the effects of wound dressing, in all patients, a sterile and transparent film dressing (OPSITE® Post-OP visible, Smith & Nephew Woundcare, Hamburg, Germany) will be applied on the 2nd postoperative day .

#### Requirement of analgesics and perception of pain during postoperative recovery in the hospital

Secondary objectives are the need for analgesics (type and amount of the prescribed ones and those beyond) during post-operative recovery and the individual perception of pain. The information will be gathered by chart review, interview, and utilization of a numeric analog scale (NAS), respectively. The latter is scaled from zero (no pain at all) to ten (maximum pain) and the patient is asked to rank the perceived pain accordingly.

#### Recurrence of mobility during postoperative recovery and follow-up

Clinically common goniometers will be used to gain knowledge on the mobility (ROM) of the affected and contralateral ankle joint (dorsi- and plantarflexion) at the time of discharge from the hospital and during follow-up according to the neutral zero method.

These investigations are paralleled by an assessment of the functional outcome, i.e., the walking abilities. For this purpose, pressure-sensitive insoles (loadsol® system, novel GmbH, Munich, Germany) providing time-resolved information on normal ground reaction forces will be used. The patients are provided with the system at the time of discharge and during follow-up visits and will use it with or without assistive devices, as appropriate. Every session will start with a familiarization period followed by a recording of time-resolved normal ground reaction forces during 3 min straight level walking at a self-selected speed. This approach will provide information on functional recovery, i.e., cadence, gait velocity, and the factor of imbalance as a surrogate marker for recurrence of gait symmetry during fracture healing.

#### Ca accrual to the bone during fracture healing

Recently, the naturally occurring stable (non-radioactive) Ca isotopes ^44^Ca and ^42^Ca have been established as markers of bone mineral balance. Both isotopes are provided by and absorbed from the diet and the rate of sequestering into different body compartments is intimately linked to the atomic mass. Thus, chemical transport reactions result in an enrichment of ^42^Ca in the respective compartment of the body, while the isotopically heavier ^44^Ca is excreted [16, 25–27]. Consequently, the δ^44/42^Ca_serum_ ratio reflects bone mineral balance, i.e., the ratio is higher under conditions of mineral accrual by the skeleton compared to a situation when bone resorption exceeds bone formation. The same holds true for the isotope ratios in urine and feces [15]. To monitor accrual of Ca during fracture healing, patients are asked to provide serum and urine samples on a voluntary basis the morning before surgical fixation of the fracture, at least once during post-operative recovery, at time of discharge from the hospital, and at the time of the follow-up visits. All samples will be taken after overnight fasting and will be stored at − 80 °C until the time of analysis, and patients will be asked to record a food frequency questionnaire before the follow-up visits [28].

#### Scoring of bone mineral density

Quantitative pulse echography (Bindex®, Bone Index Finland, Ltd., Kuopio, Finland) is used to assess bone mineral density the day before surgery, at the time of discharge, and the final visit. In particular, the cortical thickness of the healthy tibia is measured and translated into an estimate of bone mineral density (i.e., healthy, osteopenic, and osteoporotic bone) via the in-build proprietary algorithm [24, 29].

### Adverse events reporting and data safety monitoring

This is a single-center evaluator blinded randomized controlled pilot study, and the device-based application of negative pressure therapy is deemed to be of rather low risk. The same holds true for the proposed outcome measures, i.e., chart review, questionnaires, and touchless 3D optical scanning of the ankle and lower leg. However, all complications (e.g., wound-healing problems, infections) are to be recorded, and participants will be encouraged to report any discomfort related to either therapy or outcome measures. Discontinuation or modification of the treatment will be an individual decision of the responsible physician. There will be no special criterion for stopping the trial except for severe postoperative complications making a surgical reintervention necessary.

### Data collection, management, and statistical analysis

Clinical and anthropometric data including a history of disease, time to surgery, current medication, staged or single-step surgery, comorbidities, and the results of routine assessments (clinical chemistry, imaging) at baseline will be gathered by chart review and interview. Furthermore, postoperative complications (wound healing problems, non-union, infections) are recorded in both treatment groups. At the time of enrollment, patients will receive a unique identification code (ID), and all documents and hard copies generated for the purpose of this trial will be linked to this identifier. The underlying key will be stored in a password-protected file accessible only to the principal investigators (DCF, TM). Of note, the evaluator will be unblinded only after the last patient has finalized the study examinations. Regardless of this, access to the data will be limited to the researchers involved in this trial.

Data will be analyzed using the SPSS statistical package 26.0 (SPSS Inc., Chicago, IL) and Sigma Plot V10 (Systat Software GmbH, Erkrath, Germany) for graphical presentation. The normal distribution of continuous variables will be evaluated by the Kolmogorov-Smirnov test, descriptive summary measures will be calculated. Categorical variables are summarized as numbers and percentages. For comparison of the categorical variables, the chi-square test or Fisher’s exact test will be used, as appropriate. Repeated measure analysis will allow for longitudinal examination of the treatment effects and differences between the study groups. Data from all randomized participants will be included (intent-to-treat analysis). No missing data imputation will be performed, and in view of the rather small number of participants per treatment arm, neither subgroup or adjusted analyses are planned. During the study, patients and personnel are encouraged to mention all problems related to the study investigations and especially to the acceptance of the contact-less 3D optical scanning procedure. At the time of discharge, patients randomized to RICE^+^ will be asked by the one in charge for the application of the device-based negative pressure therapy to rate this additional treatment on a scale ranging from 1 (very good) to 5 (no effect). Proceeding to the main trial will be deemed feasible if (a) at least two-thirds of the patients allocated to RICE^+^ will retrospectively judge this additional treatment positive and (b) at least two-thirds of all patients accept the repetitive contact-less 3D optical scanning procedure.

## Discussion

The study is designed to assess the effects of the classical RICE concept and the additional application of a device-based negative pressure therapy (RICE^+^) with respect to the resorption of lymphedema related to the surgical fixation of an isolated ankle fracture. Although standardization of the components of RICE is hard to achieve even in a hospital setting, we record the number of ice packages per day not only as a marker of the individual preference for cooling but as a surrogate marker of adherence to the baseline therapy, as well. To objectively assess the effects of either therapeutic approach, we decided to perform touchless 3D optical scanning as this turned out to be a highly reliable technique for quantifying the volume of the foot and ankle region [14]. Given the multiple implications of ankle swelling with respect to pain, complications, mobility, and outcome, we selected a rather broad panel of outcome measures covering a 12-week postoperative interval. Although this approach might be prone to any bias related to the RICE concept and/or to the individual care after discharge from the hospital, it will certainly provide data on the immediate effects of RICE vs RICE^+^. The blinding of the evaluator and even the staff involved in surgery and postoperative care and the fact that the person in charge for the device-based application of negative pressure therapy will show up anyway are expected to minimize the risk of a bias in standard care. Further, the dynamics of the ratio of naturally occurring calcium isotopes in relation to the postoperative development of lymphedema, the manifestation of eventual postoperative complications as infection or non-union, and bone mineral density will permit insight into the mechanisms of interaction between perioperative swelling and uneventful bone remineralization or prolonged demineralization in case of complications in the postoperative period. In summary, the results of the proposed pilot study will enable us to decide whether it is worth comparing the effects of RICE and RICE^+^ in a sufficiently powered multicenter trial and which of the outcome markers are best suited for this purpose. Without saying, this decision will consider the feedback of patients on either therapeutic approach as well as comments from the staff.

### Ethics and dissemination

The protocol was approved by the institutional Ethics Committee (Rostock University Medical Center, Rostock, Germany) in accordance with the Declaration of Helsinki (approval number: A 2020-0092). The results of this study will be actively disseminated through scientific publications and conference presentations.

### Trial registration

DRKS Identifier 00023739 version 1, posted on December 3, 2020. Recruitment is scheduled from June 2021 to June 2022.

### Protocol amendments

Any changes to the protocol will be presented for approval to the university’s ethics committee. Subsequently, the revised version of the protocol will be deposited at the DRKS - German Clinical Trials Register (https://www.dimdi.de/dynamic/de/weitere-fachdienste/deutsches-register-klinischer-studien/studienregistrierung/).

### Collection of biological specimens

This trial involves the voluntary provision of blood and urine samples for additional biochemical analysis of markers related to bone metabolism and mineralization.

## Data Availability

No datasets were used or analyzed in this manuscript. Details about the procedures and information materials handed to the participants will be provided on reasonable request.
